# Disordered eating behaviour in young adults with type 1 diabetes mellitus

**DOI:** 10.1186/s40337-018-0194-2

**Published:** 2018-05-02

**Authors:** S. Keane, M. Clarke, M. Murphy, D. McGrath, D. Smith, N. Farrelly, S. MacHale

**Affiliations:** 1 Department of Psychiatry, Beaumont Hospital, Beaumont Road, Dublin 9, Ireland; 2 Trinity College Health Service, Dublin, Ireland; 3 Department of Endocrinology, Beaumont Hospital, Beaumont Road, Dublin 9, Ireland

**Keywords:** Eating disorders, Primary health care, Eating disorder examination questionnaire (EDE-Q), Disordered eating behaviour (DEB), diabetes mellitus

## Abstract

**Background:**

The combination of eating disorders and diabetes is associated with increased risk of morbidity and mortality. The aim of this study is to compare the prevalence of disordered eating behaviour (DEB) in young adults with type 1 diabetes mellitus to a sample of non-diabetic controls, and to examine the relationship of DEB to glycaemic control.

**Methods:**

The Eating Disorder Examination Questionnaire (EDE-Q) was administered to 51 individuals aged 18–30 years attending an outpatient diabetic clinic in a large university teaching hospital. Glycaemic control was assessed by the glycosylated haemoglobin (HbA1c). The control group comprised a consecutive sample of 236 male and female students aged 18–30 years attending a university primary health care service.

**Results:**

The mean global EDE-Q score for the diabetes group was 0.82 ± 1.1 (mean ± SD) and the mean for the control group was 1.4 ± 1.3 (mean ± SD). The diabetes group was significantly more likely to have a lower global EDE-Q score compared to the control group. There was no association between the global EDE-Q score of the diabetes group and HbA1c level.

**Conclusions:**

We did not find increased levels of disordered eating behavior (DEB) in young adults with type 1 diabetes mellitus compared to a non-diabetic control sample.

## Plain English Summary

Eating disorders in individuals with diabetes results in acceleration of disease related complications. There is conflicting information in the literature regarding the prevalence of disordered eating behaviour in diabetes. In this study we examined the rates of disordered eating behaviour in a young adult sample with an established diagnosis of type 1 diabetes and compared them to a young adult control sample without diabetes. We also examined the association between the level of blood sugar control and the level of disordered eating in the diabetic sample. 274 participants were included in this study, 49 of whom had a diagnosis of type 1 diabetes. We did not find increased rates of disordered eating behaviour in our diabetic sample. Our findings highlight the importance of all clinicians, in both primary and secondary care settings, being aware of the presentation of eating disorders in young adults.

## Background

The treatment of young adults with type 1 diabetes mellitus (T1DM) is often complicated by comorbid eating disorders (ED) and disordered eating behaviour (DEB). To date studies have shown varying prevalence rates of eating disorders in individuals with T1DM [1]. A 2005 meta-analysis found no significant difference in prevalence of Anorexia Nervosa in T1DM compared to controls [2]. DEB comprises a wide range of eating disorder pathology including dietary restriction, binge eating, and compensatory behaviours for the purpose of weight control, ranging from subclinical to clinical eating disorders. Compensatory behaviours include self-induced vomiting, laxative misuse and excessive exercise. The combination of diabetes with DEB is of particular concern due to its association with poor glucose control and accelerated onset of diabetes related complications [3, 4]. Retinopathy has been reported in 86% of young women, with a 7±4 year duration of diabetes, who had highly disordered eating at baseline on 4-year follow-up [3]. Moreover the coexistence of eating disorders and diabetes is frequently associated with non-compliance with treatment for diabetes and the abuse of insulin to promote weight reduction [5]. “Diabulimia” is a term increasingly used to refer to this intentional restriction or omission of insulin for weight control [6, 7]. Deliberate insulin omission is a long recognized cause of recurrent diabetic ketoacidosis in adolescents with T1DM [8]. Approximately one-third of people with T1DM intentionally omit insulin [9].

It has been hypothesized that features of T1DM may predispose vulnerable individuals to eating disorders [4]. These features include the dietary restraint necessary for management of diabetes, the cycle of weight loss at disease onset and subsequent weight gain with initiation of insulin, the trend towards higher body mass index (BMI), and the option of misuse of insulin to influence body weight. Negative coping strategies and mood disturbances may increase the risk of developing co-morbid eating disorders in individuals with T1DM [10].

Studies to date in this area have produced conflicting results in large part due to major variations in study design. Some studies show an increased risk of DEB/ED in patients with diabetes compared with the general population [4, 11] while other studies do not report an increased risk [12–14]. Studies comparing the frequency of DEB/ED in males with and without diabetes are scarce [15]. A systematic review and meta-analysis found DEB and ED to be significantly more common in type 1 diabetic adolescents than in their peers [16]. However this difference was found to be no longer significant when restricting the analysis to studies using diabetes adapted assessment tools. Diabetes adapted tools identify eating disorder behaviours that are unique to T1DM, such as the underuse or omission of insulin for the purpose of weight loss.

Eating disorders in individuals with T1DM are significant and are associated with serious morbidity [3] and worse treatment outcomes [7]. We aimed to further explore this comorbidity by comparing the prevalence of disordered eating behaviour in a young adult sample with T1DM to a control sample in an Irish student population using the EDE-Q, a detailed questionnaire used to identify probable cases of eating disorders.

## Method

The Eating Disorder Examination Questionnaire (EDE-Q) version 6.0 was used in this study. The EDE-Q is a 28 item instrument which focuses on the previous 28 days and measures core eating disorder behaviours. The EDE-Q produces a global score and four subscale scores: restraint, shape concern, weight concern and eating concern.

The global score is the average of the four subscale scores. Responses are rated on a seven-point Likert scale, and higher scores indicate greater disordered eating pathology. A cut-off score of ≥4 is commonly used to indicate clinical significance [17–20].

The frequency of key eating and compensatory behaviours such as objective binge episodes, self-induced vomiting, laxative misuse and excessive exercise are also recorded. Regular occurrence of excessive exercise was defined as exercising “in a driven or compulsive way as a means of controlling your weight, shape or amount of fat, or to burn off calories” (EDE-Q 6.0, item 18) for ≥20 times over the past 28 days. For regular occurrence, dietary restraint was defined as going “for long periods of time (8 waking hours or more) without eating anything at all in order to influence your shape or weight” (EDE-Q 6.0, item 2) for more than three times per week (≥13 times) over the past 28 days. For all other behaviours regular occurrence was defined as four or more occurrences over the past 28 days.

### Participants

Patients were recruited while attending an outpatient diabetic clinic for young adults in Beaumont Hospital, a large university teaching hospital in North Dublin. Inclusion criteria were individuals aged between 18 and 30 years with a diagnosis of T1DM. A non-consecutive sample of 51 individuals (females *n* = 20, males *n* = 31) with diabetes were recruited. One male individual was excluded as he did not complete the consent form and another male because he failed to respond to over half of the items, leaving a total of 49 participants (females *n* = 20, males *n* = 29). The EDE-Q was used to assess eating disorder psychopathology. Glycaemic control in the diabetic sample was assessed by the glycosylated haemoglobin (HbA1c) measurement nearest to the date of assessment. [All, bar three, HbA1c measurements were taken within one month of the assessment. Of the HbA1c assessments that were not taken within one month, one was taken five months prior to the assessment, one four months prior and one three months prior]. None of our diabetic sample were on mixed insulin, 15% were on insulin pump therapy and the remainder were on a basal bolus regime.

For the control group the EDE-Q was administered to a consecutive sample of 236 male and female students attending a university primary health care service over a one month period. Students with T1DM or known history of chronic illness were excluded. Ten students did not consent to the study and one student did not complete the questionnaire leaving a total of 225 participants (females *n* = 190, males *n* = 35). Written informed consent was obtained from all participants. The study was reviewed and approved by the Ethics Committees of Beaumont Hospital and of Trinity College, Dublin.

### Statistical analysis

The scoring methods advised by Fairburn and coauthors [21] were used for the calculation of EDE-Q subscale and global scores. With respect to the diabetic group, one participant failed to respond to over half of the items and so was excluded from the analysis. There were no other participants with more than one missing item.

With respect to missing data for the control group, 0.3% of the items required to score the EDE-Q subscales were missing. One participant failed to respond to three of the five eating concern questions and so their eating concern subscale was excluded from the analysis. Another participant failed to respond to two items but, otherwise, there were no other participants with more than one missing item. Any missing responses were replaced with the mean item score as done in previous studies using this instrument. Differences between groups were initially examined using chi-square tests for categorical variables and t-tests for continuous variables. Univariate analyses for binary outcomes were then carried out using logistic regression with 95% confidence intervals. Confounders were adjusted for in multivariate logistic regression models. Univariate and multivariate linear regression models were used for continuous outcomes. In the univariate model (unadjusted) the outcome or dependent variable was diabetes and the independent or risk factor was eating behavior. In the multivariate models BMI and gender were added as additional independent variables. All analyses were carried out using STATA, version 13.

## Results

There were 49 participants with diabetes and 225 control participants without diabetes. There were significantly more females in the control group compared to the diabetes group (x^2^ (1, *N* = 274) = 42.8, *p* < .0001). 84.4% of controls were female and 40.8% of the diabetes group were female. There was no difference in age between both groups (*t*(259) = 0.98, *p* = =0.32) The mean age of those with diabetes was 21.4 ± 2.5 (mean ± SD) years (range: 26–18 years) and the mean age for the controls was 22 ± 4.1 (mean ± SD) years (range: 58–18 years). The mean BMI for the diabetes group was 23.5 ± 3.8 (mean ± SD) kg/m2 (range: 17.6–34.1) and the mean BMI for controls was 22.3 ± 3.5 (mean ± SD) kg/m2 (range: 15.4–39.9). Within the diabetes group 28.9% had a BMI > 25 kg/m2 compared to 14.5% of the control group. However there was no significant difference in BMI between the groups when adjusted for gender (OR = 1.06, 95%CI: 0.9–1.1, *p* = 0.18).

### EDE-Q scores

The diabetes group was significantly more likely to have a lower global EDE-Q score compared to the control group (OR = 0.6, 95%CI: 0.4–0.8, *p* < 0.00). The mean global EDE-Q score for the diabetes group was 0.82 ± 1.1 (mean ± SD) and the mean for the control group was 1.4 ± 1.3 (mean ± SD). The difference was still significant when adjusted for gender and BMI (OR = 0.6, 95%CI: 0.4–0.9, *p* < 0.05). There were no significant changes in the odds ratios when adjusting BMI and gender either together or individually (adjusting for gender only OR = 0.7, 95% CI: 0.5–1.1, p < 0.05) (adjusting for BMI only OR = 0.5, 95% CI: 0.3–0.9, *p* < 0.05). This pattern was similar for the subscales of the EDE-Q. The diabetes group had significantly lower scores on weight concern (OR = 0.7, 95%CI: 0.5–0.9, *p* < 0.05) and shape concern (OR = 0.7, 95%CI: 0.6–0.9, *p* < 0.05); both adjusted for gender and BMI. There was also a trend for lower scores for the diabetes compared to the control group on the restraint and eating-concern subscales (OR = 0.7, 95%CI: 0.5–0.8, *p* = 0.07, OR = 0.6, 95%CI: 0.4–1.1, *p* = 0.08, respectively; both adjusted for gender and BMI). See Table 1.

**Table 1:** Eating Disorder Examination Questionaire (EDE-Q) global and subscale scores

EDE-Q	Diabetic Group (*n* = 49)	Control Group (*n* = 225)
	*Means (S.D*)	*Means (S.D)*
Global Score	0.82 (1.1)	1.4 (1.3)
Restraint	0.7 (1.1)	1.2 (1.3)
Eating Concern	0.4 (0.6)	0.8 (1.1)
Shape Concern	1.2 (1.6)	2.1 (1.6)
Weight Concern	0.9 (1.2)	1.7 (1.6)

5.3% of controls had an EDE-Q score in the clinical range; no individuals in the diabetes group had a score in this range. 4.8% of the female controls engaged in purging behaviour with 2.1% of the sample engaging in regular self-induced vomiting. 2.1% of the female control group admitted to laxative misuse with 1.6% of the sample admitting to regular laxative misuse. None of the diabetic females engaged in any purging or laxative misuse behaviours. Both female diabetic and control groups engaged in similar amounts of objective binge episodes. Overall, when combining for gender, 14.3% of the diabetic group and 16% of the control group engaged in regular binging behavior. 5% of the female diabetic group admitted to regular excessive exercise compared to 1.1% of the female control group. See Table 2.

**Table 2:** Proportion of women engaging in any or regular occurrence of key eating and compensatory behaviours

Key Behaviour	Any Occurrence (%)		Regular Occurrence (%)	
	Diabetic N = 20	Control N = 190	Diabetic N = 20	Control N = 190
Objective Binge Episodes (OBE)	40	31.5 *p = 0.441*	20	15.8 *p = 0.410*
Self-induced Vomiting	0	4.8 *p = 0.395*	0	2.1 *p = 0.665*
Laxative Misuse	0	2.1 *p = 0.665*	0	1.6 *p = 0.737*
Excessive Exercise	30	30.5 *p = 0.965*	5	1.1 *p = 0.264*

2.9% of the male controls engaged in purging behaviour, which was comparable to 3.4% of the male diabetics. None of the male controls or diabetics engaged in any regular self-induced vomiting. Table 3 shows the proportion of men engaging in any or regular occurrence of key eating and compensatory behaviours. There were no statistically significant differences in the key behaviors, whether any occurrence or regular. Statistical testing was done with a chi-square test in cases where the number of variables in each cell exceeded five, and with a Fisher’s Exact test when it did not.

**Table 3:** Proportion of men engaging in any or regular occurrence of key eating and compensatory behaviours

Key Behaviour	Any Occurrence (%)		Regular Occurrence (%)	
	Diabetic N = 29	Control N = 35	Diabetic N = 29	Control N = 35
Objective Binge Episodes (OBE)	20.7	22.9 *p = 0.835*	10.3	17.1 *p = 0.342*
Self-induced Vomiting	3.4	2.9 *p = 0.705*	0	0
Laxative Misuse	3.4	0 *p = 0.453*	0	0
Excessive Exercise	31	14.3 *p = 0.107*	0	2.9 *p = 0.547*

### HbA1c level

The mean HbA1c level for the diabetes group was 76 ± 23.3 (mean ± SD) mmol/mol (9.1%). 14.9% of the sample had a HbA1c of < 53 mmol/mol (7.0%). 6.4% had a reading of between 53 mmol/mol < 58 mmol/mol (7.0 < 7.5%). 6.4% had a reading of between 58 mmol/mol < 64 mmol/mol (7.5 < 8.0%). 27.7% had a reading of between 64 mmol/mol < 75 mmol/mol (8.0 < 9.0%). 12.8% had a reading of between 75 mmol/mol < 86 mmol/mol (9 < 10%) and 31.9% of the sample had a reading of ≥ 86 mmol/mol (10%). There was no association between the global EDE-Q score of the diabetes group and HbA1c level; there was also no association between any of the EDE-Q subscale scores and HbA1c level. Individuals with diabetes who had a BMI of < 18.5 (*N* = 18) had a significantly lower HbA1c level compared to those with a healthy BMI between 18.5 and 25 (*β* = 39.1, *t*(42) = 2.3, *p =* 0.05; adjusted for gender). There was no difference in HbA1c level between those with a BMI of over 25 and those with a healthy BMI. There was also no difference in HbA1c level across ages in the diabetes group.

## Discussion

The EDE-Q results of our study did not show an increased prevalence of DEB in a young adult sample with T1DM compared to a student control sample. The diabetic group had significantly lower global EDE-Q scores compared to the control sample, which was still significant when adjusted for gender and BMI. This pattern was similar for each of the individual subscales of the EDE-Q, also adjusted for gender and BMI. Although methodologically different, our results concur with the lower adjusted prevalence of DEB in patients with diabetes compared with a comparison group of other studies [13, 14].

Interestingly, none of the female diabetics engaged in any serious compensatory behaviours such as self-induced vomiting or laxative misuse. A potential confounder to consider is that having the option of insulin restriction for the purpose of weight control could result in reduction of other purging behaviours in individuals with diabetes. This may have biased our results as we did not adapt the EDE-Q for individuals with diabetes and we did not assess the diabetic specific DEB of insulin purging. The Diabetes Eating Problem Survey (DEPS-R) is a 16 item, self-report, screening measure for assessing disordered eating in diabetes which has demonstrated internal consistency and construct validity and may be a useful screening tool in this population [22]. Also, our diabetic sample consisted of a young adult group attending a secondary care endocrine outpatient clinic with access to multidisciplinary support, including dietician input.

The mean HbA1c level for the diabetic group was 76 mmol/mol (9.1%). There was no association between the global EDE-Q score of the diabetes group and the HbA1c level. There was also no association between any of the EDE-Q subscale scores and HbA1c level. However, as none of the diabetic group scored within the clinical range on EDE-Q this result should be interpreted with caution.

Research in this area has been conflicting due to huge variation in methodological approaches used to date and the lack of standardised, universally accepted assessment tools for the detection of DEB. We used a detailed questionnaire, the EDE-Q, which has shown good psychometric properties and can be used to identify probable cases of eating disorders [20]. The EDE-Q is the self-report version of the Eating Disorder Examination (EDE), which is widely considered the instrument of choice for the assessment and diagnosis of DSM eating disorders [23]. Studies have shown a high level of agreement between the EDE-Q and the EDE interview in the assessment of attitudinal features of eating disorder psychopathology [20].

This study has a number of limitations. First, the relatively small sample of young adults with diabetes recruited limited power to identify differences between the samples. We acknowledge the possibility of type II error in our findings. It is important to consider that our methods of recruitment (i.e: a non-consecutive diabetic sample and a consecutive control sample) may have also influenced our findings as individuals with eating disorders have a tendency to conceal their illness and avoid professional help [24, 25].

A further limitation is that, as the control group was taken from a third level university sample, it is likely to contain a greater prevalence of high achieving perfectionistic individuals of a different sociodemographic background and hence is not generalizable to the general young adult population. Additionally, the control sample was recruited from individuals attending a primary care service for non-eating disorder related difficulties and, as such, may represent a false high prevalence compared to those who do not access health care services. However, it is worth noting that the mean global EDE-Q score for the female component of our control group (1.51) was comparable to mean global scores obtained in previous studies of an English community based sample (1.55) as well as a Swedish (1.56) and an Australian (1.52) general population sample [26]. Also, the EDE-Q is a self-report measure which is not a diabetic specific assessment tool and does not ask about the manipulation or omission of insulin to influence weight.

## Conclusions

We did not find increased levels of disordered eating in a non-consecutive outpatient sample of young adults with T1DM compared to a non-diabetic control group. A limitation of our study is that we did not use a screening tool that was adapted for individuals with T1DM. Studies are needed to determine risk factors for disordered eating in people with diabetes over and above the presence of diabetes. Early identification and intervention is crucial given the risk of increased morbidity and mortality associated with the combination of eating disorders and diabetes [1]. Future studies should focus on the identification of patients with comorbid DEB and T1DM using diabetic specific screening instruments, the development of preventative and treatment interventions, and the integration of these interventions into mainstream diabetic care.

## Abbreviations

BMI: Body mass index
CI: Confidence interval
DEB: Disordered eating behavior
ED: Eating disorders
EDE: Eating Disorder Examination.
EDE-Q: Eating Disorder Examination Questionnaire
HbA1c: Glycosylated haemoglobin
OR: Odds ratio
SD: standard deviation
STATA: syllabic abbreviation of the words statistics and data (the name of a statistical software package)
T1DM: Type 1 diabetes mellitus
